# Noninvasive assessment of liver fibrosis can predict clinical outcomes at late follow-up after a sustained virological response in HCV patients?

**DOI:** 10.1016/j.clinsp.2024.100381

**Published:** 2024-05-10

**Authors:** Taisa Grotta Ragazzo, Patricia Momoyo Yoshimura Zitelli, Daniel F. Mazo, Claudia P. Oliveira, Flait José Carrilho, Mário Guimarães Pessoa

**Affiliations:** a Divisão de Gastroenterologia e Hepatologia Clínica, Departamento de Gastroenterologia, Hospital das Clínicas, Faculdade de Medicina, Universidade de São Paulo ((HCFMUSP), São Paulo, SP, Brazil; b Divisão de Gastroenterologia (Gastrocentro), Faculdade de Ciências Médicas, Universidade Estadual de Campinas (UNICAMP), Campinas, SP, Brazil

**Keywords:** Hepatitis c chronic, Elasticity imaging techniques, Liver Cirrhosis

## Abstract

**Objectives:**

The primary objective was to evaluate Liver-Related Events (LREs), including hepatic decompensation (ascites, hemorrhagic varices and encephalopathy) and Hepatocellular Carcinoma (HCC), as well as changes in liver stiffness during the follow-up period among patients who achieved a Sustained Virological Response (SVR) after treatment for chronic Hepatitis C Virus (HCV) infection.

**Methods:**

A total of 218 patients with HCV were treated, and those who achieved an SVR were followed up for 3-years. Transient Elastography (TE) using FibroScan® was performed at various time points: before treatment, at the end of treatment, at 6-months post-treatment, at 1-year post-treatment, at 2-years post-treatment, and at 3-years post-treatment.

**Results:**

At 6-months post-treatment, a Liver Stiffness Measurement (LSM) cutoff of > 19 KPa was identified, leading to a 14.5-fold increase in the hazard of negative outcomes, including decompensation and/or HCC. The analysis of relative changes in liver stiffness between pre-treatment and 6-months posttreatment revealed that a reduction in LSM of -10 % was associated with a -12 % decrease in the hazard of decompensation and/or HCC, with this trend continuing as the LSM reduction reached -40 %, resulting in a -41 % hazard of decompensation and/or HCC. Conversely, an increase in the relative change during this period, such as an LSM increase of +10 %, led to *a* + 14 % increase in the hazard of decompensation. In cases where this relative change in LSM was +50 %, the hazard of decompensation increased to +92.

**Conclusion:**

Transient elastography using FibroScan® can be a good tool for monitoring HCV patients with SVR after treatment to predict LREs in the long term.

## Highlights

• Transient elastography is the best accepted noninvasive technique for evaluating fibrosis in patients with HCV.
• The variation in the LSM measurement can demonstrate the risk of liver-related events.
• The presence of LSM ≥19 kPa 6 months after treatment increases the risk of liver-related events.

## Introduction

Since 2013, the landscape of hepatitis C treatment has undergone major advancements with the advent of novel Direct-Acting Antivirals (DAAs). With cure rates exceeding 90 %, the World Health Organization established guidelines aiming to eliminate hepatitis C by 2030.^1^ However, the COVID-19 pandemic disrupted all facets of hepatitis treatment.^2^ Currently, an estimated 58 million individuals are affected by the hepatitis C virus worldwide, which resulted in approximately 290.000 HCV-related deaths in 2019.^1^^,^^3^ HCV is recognized as the primary cause of cirrhosis, and Hepatocellular Carcinoma (HCC) and serves as one of the leading indications for Liver Transplantation (LTx).^4^ An estimate conducted to predict the incidence of decompensated cirrhosis related to hepatitis C virus infection in 2030 indicated an estimated increase from 148.000 cases in 2020 to 174.000 cases worldwide.^5^ Viral hepatitis continues to be the dominant etiology of liver disease associated with HCC in Latin America. Improvements in healthcare accessibility, surveillance, and ongoing medical education are imperative. Latin America witnesses an estimated annual occurrence of over 38,000 HCC cases.^6^ Thus, despite the availability of an HCV cure, the ongoing emergence of HCC remains a subject of investigation. Recent studies have examined the role of Non-Invasive Tests (NITs) in managing patients with advanced fibrosis after a Sustained Virological Response (SVR) to stratify the risk of Liver-Related Events (LREs).^7^ Transient Elastography (TE) using FibroScan® (Paris, France) and other NITs such as AST- to-Platelet Ratio Index (APRI) score and Fibrosis-4 (FIB4) score, are frequently employed for patient evaluation and treatment decision-making.^8^ Studies focusing on patients with HCV-related cirrhosis who achieve SVR following DAA therapy assessed the risk of HCC by comparing Liver Stiffness Measurement (LSM) at the beginning and end of treatment. For instance, one study evaluated 139 cirrhotic patients with SVR over a 15-month period and found that a reduction of less than 30 % in the LSM was an independent risk factor for HCC development.^9^ The initial decline in LSM values is attributed to regression of inflammation; however, fibrosis persists.^10^ Moreover, research has suggested the presence of markers indicating an altered balance between collagen degradation and formation after viral clearance, suggesting favorable effects on liver fibrosis.^11^ Given the importance of this topic, the present study's objective was to observe the progression of patients over a 3-year period post-SVR and explore the relationship between changes in liver stiffness and the development of hepatic decompensation (LREs) and HCC.

## Materials and methods

This prospective cohort study was conducted at the Gastroenterology and Hepatology Service of the Department of Gastroenterology at Hospital das Clínicas of the University of São Paulo School of Medicine (FMUSP) between December 2015 and December 2017. A total of 235 patients were enrolled in the study. The inclusion criteria were individuals aged ≥18-years with chronic hepatitis C (HCV RNA positive) who underwent Direct-Acting Antiviral (DAA) treatment. The exclusion criteria were patients with HIV or Hepatitis B Virus (HBV) coinfection, current or previous hepatic decompensation (ascites, hemorrhagic esophageal varices and encephalopathy) and/or HCC, noncirrhotic portal hypertension (schistosomiasis), those who declined to provide informed consent, and individuals who did not achieve SVR. The laboratory data used for analysis and the calculations of APRI, FIB4, Child-Pugh, and Meld scores were retrieved from the patient's electronic medical records. For statistical analysis, two patients diagnosed with HCC before the third visit were excluded. TE was performed by a single experienced operator using the FibroScan® MODEL 502 device (Echosens, Paris, France) on six different occasions: pretreatment (V1), end of treatment (V2), 6-months (V3), 1-year (V4), 2-years (V5), and 3-years (V6) after treatment. Patients were categorized based on their LSM values at the outset into three groups: Group A with LSM < 9.5 KPa (76 patients); Group B with LSM ≥ 9.5 KPa and < 14.5 KPa (59 patients); and Group C with an LSM ≥14.5 KPa (83 patients). All examinations comprised ten valid measurements, with an Interquartile Range (IQR/M) < 30 % or a success rate > 60 %, as recommended by current guidelines.^12^ Drug regimens and treatment durations (12 or 24 weeks) were determined by the patient's physicians in adherence with the Brazilian Ministry of Health's HCV treatment protocol at the time of the conduction of the study.^13^ Lifestyle habits, including self-reported smoking and alcohol consumption, were documented, as was the presence of diabetes and hypertension (defined by the use of at least one medication) and the presence of metabolic syndrome (as per NCEP-ATP III criteria).^14^ During the follow-up period, hepatic decompensation events such as Ascites (ASC), Hepatic Encephalopathy (HE), Variceal Hemorrhage (VE), and HCC, collectively referred to as Liver-Related Events (LREs), were monitored following confirmation of SVR. Additionally, deaths that occurred during the study period, particularly those attributed to liver-related causes, were recorded.

### Ethical considerations

The study was performed in accordance with the principles of the Declaration of Helsinki. The protocol was approved by the institutional ethics board (number 1,553,607), and written informed consent was obtained from all participants.

### Statistical analysis

Exploratory data analysis involved calculating the means, medians, standard deviations, and ranges for continuous variables, while categorical variables were summarized using frequencies and proportions. The normal distribution of continuous variables was assessed for skewness, kurtosis, and the Shapiro-Wilk test. Comparison of continuous variables between groups was carried out utilizing Student's *t*-test. To compare absolute and relative changes in fibrosis values over time, the Friedman test was employed, with multiple comparisons adjusted using the Bonferroni method when applicable. Correlation analysis was conducted using the Spearman correlation coefficient.

The decompensation-free/HCC-free survival curve was constructed using the Kaplan-Meier method. The time between the first visit and the occurrence of decompensation and/or HCC or censoring was calculated. Cox regression was used to analyze predictors of decompensation and/or HCC development. Correction of regression beta coefficients was carried out using the method proposed by Firth due to the unbalanced nature of the data in this study.

The optimal cutoff point for continuous predictors in the Cox regression analysis was determined based on the survival curve distribution. This method yields a cutoff value associated with the most significant relationship with the survival curve. The significance level for the tests was set at 5 %. Statistical analysis was conducted using IBM SPSS Statistics software version 28 (IBM Corporation, NY, USA)^15^ and the survminer package of the R program (R CORE TEAM, 2015).^16^

## Results

**Fig. 1:**
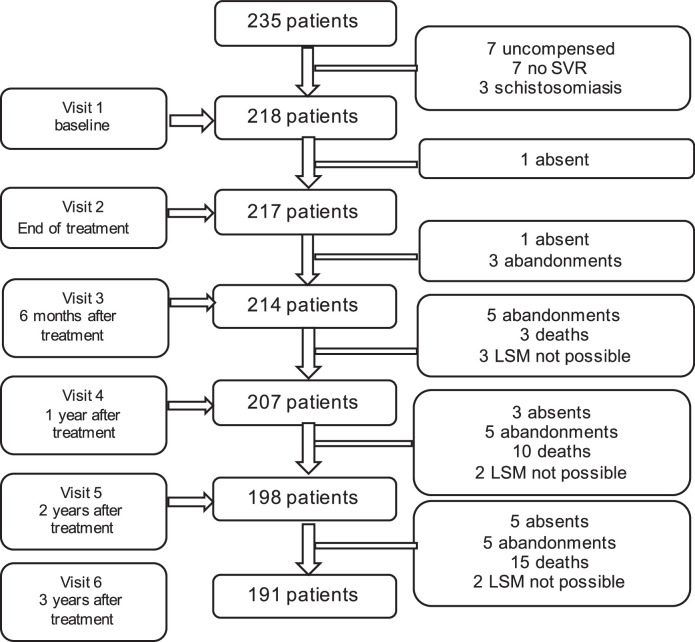
Patient distribution during the 3-year follow-up post-Sustained Virologic Response (SVR).

Initially, 235 patients were enrolled in this study, but 17 individuals were subsequently excluded. Seven patients failed to achieve an SVR, seven patients presented initial decompensation at the first visit, and three patients had portal hypertension secondary to schistosomiasis. Consequently, this study followed up 218 patients for 3-years after treatment. The distribution of patients over 3-years of follow-up at each visit, the complications, the onset of decompensation and/or HCC, and the total number of patients included in the statistical analysis are shown in Fig. 1 below.

Among these 218 patients, 63.3 % were female, with a mean age of 59.38 years, and 61.9 % were of Caucasian origin. Regarding self-reported lifestyle habits, 14.2 % were smokers, and 15.6 % consumed alcohol (≥ 40 g/day for women and ≥ 30 g/day for men).^17^ The preexisting comorbidities included diabetes mellitus in 32.6 %, hypertension in 57.5 %, and metabolic syndrome in 40.4 %. Regarding HBV serology, 93 patients (42.7 %) tested positive for isolated anti-HBs, 15 patients (6.9 %) exhibited isolated anti-HBc IgG, and in 31 patients (14.2 %), both markers were present. Concerning HCV treatment, 55.1 % had previously received treatment.

Over the course of the 3-year study period, 15 deaths were recorded. These deaths encompassed 4 linked to hepatic-related causes, 6 attributed to infectious etiologies (including 2 as a result of COVID-19), one due to acute pancreatitis, 2 stemming from neoplastic diseases not related to the liver, one resulting from a stroke, and another originating from a cardiac cause.

Table 1 presents the clinical and laboratory characteristics of the included patients. One hundred ninety-three patients did not exhibit hepatic decompensation or progressed to HCC. However, seventeen patients experienced hepatic decompensation, and 10 patients developed HCC.

**Table 1:** Mean baseline characteristics of patients stratified by the presence or absence of complications.

	All patients (*n* = 218)	Without complication (*n* = 193)	Decompensated (a*n* = 17)	HCC (a*n* = 10)	Decompensated + HCC (a*n* = 25)
**Age (years)**	59.38	59.08	59.29	64.6	61.28
**Female**	138 (63.3 %)	123 (63.7 %)	8 (47.0 %)	9 (90 %)	15 (60 %)
**Male**	80 (36.7 %)	70 (36.3 %)	9 (53 %)	1 (10 %)	10 (40 %)
**Ethnicity, n (%)**					
Caucasian	135 (61.9 %)	117 (60.6 %)	15 (88.3 %)	5 (50 %)	18 (72 %)
African descent	79 (36.2 %)	72 (37.4 %)	2 (11.7 %)	5 (50 %)	7 (28 %)
Asian	4 (1.9 %)	4 (2.0 %)	‒	‒	‒
BMI, Kg/m^2^, mean	27.12	27,17	26.22	26.41	26.7
**Comorbidities, n (%)**					
Diabetes mellitus	71 (32.6 %)	62 (32.1 %)	5 (29.4 %)	4 (40 %)	9 (36 %)
Hypertension	125 (57.3 %)	108 (55.9 %)	10 (58.8 %)	7 (70 %)	17 (68 %)
Metabolic sd.	88 (40.4 %)	79 (40.9 %)	5 (29.4 %)	4 (40 %)	9 (36.0 %)
Alcoholism	34 (15.6)	31 (16.0 %)	3 (17.6 %)	1 (10 %)	3 (12 %)
Smoking	31 (14.2 %)	25 (12.9 %)	3 (17.6 %)	4 (40 %)	6 (24 %)
**Hepatitis B, n (%)**					
Anti-HBc	46 (21.1 %)	38 (19.69 %)	6 (35.2 %)	3 (30 %)	8 (32 %)
Anti-HBs	124 (56.8 %)	111 (57.5 %)	10 (58.8 %)	4 (40 %)	13 (52 %)
**HCV genotype, n (%)**					
1	179 (82.1 %)	161 (83.4 %)	11 (64.7 %)	8 (80 %)	18 (72 %)
2	2 (0.9 %)	2 (1.1 %)	‒	‒	‒
3	36 (16.5 %)	29 (15.0 %)	6 (35.3 %)	2 (20 %)	7 (28 %)
4	1 (0.5 %)	1 (0.5 %)	‒	‒	‒
**Previous treatment, n (%)**					
0	98 (44.9 %)	90 (46.6 %)	8 (47.1 %)	1 (10 %)	8 (32 %)
1	75 (34.4 %)	63 (32.6 %)	7(41.2 %)	6 (60 %)	12 (48 %)
2	31 (14.2 %)	26 (13.5)	2 (11.7 %)	3 (30 %)	5 (20 %)
3	11 (5.1 %)	11 (5.7 %)	‒	‒	‒
4	2 (0.9 %)	2 (1.1 %)	‒	‒	‒
5	1 (0.5 %)	1 (0.5 %)	‒	‒	‒
**Laboratory test**					
Creatinine, mg/dL	1.37	1.36	1.81	0.78	1.48
Total bilirubin, mg/dL	0.37	0.34	0.70	0.39	0.58
ALT, U/L	66.32	65.15	68.94	85.80	75.32
AST, U/L	63.49	60.56	80.06	99.00	86.04
Albumin, mg/dL	4.20	4.24	3.82	3.93	3.88
Platelet count, mil/mm^3^	159.99	166.78	85.19	124.10	105.38
INR	1.14	1.12	1.29	1.21	1.25
Alpha-fetoprotein, ng/mL	22.14	19.89	13.94	65.33	36.88
MELD score mean	8.8	8.49	13.27	8.2	11.26
APRI score mean	1.71	1.53	3.34	3.61	3.13
FIB 4 Score mean	3.91	3.49	7.95	8.18	7.29
FibroScan®. mean	15.36	13.71	29.56	24.51	28.15
Group A	7.22	7.22	‒	‒	‒
Group B	11.77	11.66	12.57	12.17	12.92
Group C	25.38	23.39	33.21	29.80	31.96

Continuous variables are presented as medians, and categorical variables are presented as numbers (percentages).

a Two patients presented decompensation and CHC.

Initially, there was a good correlation between the NITs, as shown in Table 2. However, throughout this study, there was no regularity between the 3 methods shown in Table 3.

**Table 2:** Correlation between noninvasive markers of fibrosis (FibroScan®, APRI and FIB4).

	Spearman correlation			
	r_s_	95 % CI	p-value	r_s_^2^
**FibroScan® × APRI**	0.564	0.458; 0.655	**<0.001**	0.318
**FibroScan® × FIB4**	0.576	0.471; 0.664	**<0.001**	0.331
**APRI × FIB4**	0.920	0.891; 0.941	**<0.001**	0.846

r_s_, Spearman correlation coefficient; CI, Confidence Interval; r_s_^2^, Coefficient of determination.

**Table 3:** Comparative analysis of noninvasive markers of fibrosis between visits.

	Average values					
	Pre treatment	End of treatment	6-months post treatment	1-year post treatment	2-years post treatment	3-years post treatment
**FibroScan®**	15.36	13.48	12.72	12.08	10.95	10.21
**FIB4**	3.90	2.67	2.85	2.66	2.48	2.33
**APRI**	1.70	0.61	0.65	0.61	0.54	0.49

For the Friedman test, multiple comparisons (pairwise method) with significance values adjusted by the Bonferroni correction were performed, where for FibroScan® p-value < 0.001 among all visits. For APRI and FIB4, the p value was < 0.001 only between pre-treatment and end of treatment, for the other visits, the p value was 1.00.

Therefore, this study was conducted only with data obtained by TE. Patients were categorized into three groups based on cutoff values for LSM obtained via FibroScan® elastography in KPa at the pre-treatment assessment. Group A included patients with values < 9.5 KPa, indicating no or mild/moderate fibrosis, Group B included those with values ≥ 9.5 KPa and < 14.5 KPa, indicating advanced fibrosis; and Group C included patients with values ≥ 14.5 KPa, indicating cirrhosis.^18^ The mean LSM values exhibited a decrease of 36.48 % over the 3-year duration, with the most pronounced reduction occurring between the initial and second visits, amounting to 13.97 %. Analyzing these changes in values over the 3-year follow-up period, an increase in the total number of patients in Group A was noted from 76 patients to 129, accompanied by a decrease in the number of patients in Group B from 59 patients to 23 patients and in the number of patients in Group C from 83 patients to 39 patients.

Among the 25 patients who experienced an adverse outcome, 17 remained in the same group as their initial classification at the time of the event, 6 exhibited a decrease in LSM to a lower fibrosis group during the event, and 2 patients transitioned from Group B to Group C during the event. Two patients who experienced events prior to the 6-month post-treatment visit were excluded, and for patients with 2 simultaneous events, only the HCC event was considered.

Therefore, in the subset of 23 patients who had LREs, with an average occurrence of 319 days after the 6-month posttreatment visit, 9 had ASC, 8 had HCC, 5 had HE, and 1 had VH. Importantly, only 4 patients passed away prior to experiencing LREs.

Additionally, many patients underwent follow-up in 2020 when the COVID-19 pandemic began. There was a 7-month interruption in follow-up, which prevented the exact performance of the TE (FibroScan®) exam at the end of the 3-year period. This slightly extended the final follow-up duration for some patients.

According to the univariate analysis of the entire cohort of 216 patients, as detailed in Table 4, a statistically significant association with the MELD score was observed. This finding aligns with expectations, given that the MELD score is an indicator of liver disease severity.

**Table 4:** Univariate analysis by Cox regression of predictors of decompensation and/or HCC among the 216 patients with ≥ 9.5 KPa (baseline data).

Variable	Number of cases available in the analysis	Cases of Dec/HCC in the analysis	HR (95 % CI)	p-value
Male	213	23	1.281 (0.561; 2.927)	0.557
Age	213	23	1.020 (0.979; 1.063)	0.335
Smoking	213	23	1.761 (0.654; 4.745)	0.263
BMI	213	23	0.992 (0.915; 1.076)	0.848
Alcoholism	213	23	0.753 (0.223; 2.541)	0.648
Hypertension	213	23	1.791 (0.737; 4.355)	0.198
Diabetes	213	23	1.135 (0.481; 2.678)	0.772
Metabolic syndrome	213	23	0.788 (0.334; 1.858)	0.586
MELD score	112	21	1.118 (1.045; 1.197)	**0.001**
Child-Pugh B^a^	125	20	3.504(0.811; 15.142)	0.093
Alpha-fetoprotein	148	18	1.004 (0.998; 1.011)	0.225
FibroScan® pre-treatment	213	23	1.076 (1.051; 1.101)	**<0.001**
FibroScan® v1-v3 relative variation	212	23	1.012 (1.003; 1.022)	**<0.010**

a In relation to Child-Pugh A. Dec, Decompensation; HCC, Hepatocellular Carcinoma; HR, Hazard Ratio; CI, Confidence Interval; BMI, Body Mass Index; MELD, Model for End-Stage Liver Disease.

Subsequently, an assessment of the relative change in liver stiffness between the pretreatment and 6-month posttreatment intervals allowed for the estimation of risk ratios across various ranges of liver stiffness variation during these periods, as detailed in Table 5.

**Table 5:** Distribution of the relative variation of liver stiffness and its relationship to outcomes.

Relative variation FibroScan® V1-V3	Estimated HR	Relationship with decompensation
Decrease in relative variation		lesser decompensation
−40 %	0.59	−41 %
−30 %	0.68	−32 %
−20 %	0.77	−23 %
−10 %	0.88	−12 %
**Increase in relative variation**		**Greater decompensation**
+10 %	1.14	+14 %
+20 %	1.30	+30 %
+30 %	1.48	+48 %
+40 %	1.68	+68 %
+50 %	1.92	+92 %

As observed, a patient who experienced a −40 % reduction in liver stiffness between the two time periods exhibited a corresponding −41 % reduction in the hazard of decompensation and/or HCC. Likewise, a −30 % reduction in liver stiffness leads to a −32 % reduction in the hazard of decompensation and/or HCC, a −20 % reduction results in a −23 % hazard reduction, and a −10 % reduction corresponds to a −12 % hazard reduction.

Conversely, when there was an increase of +10 % in liver stiffness values between the two time periods, the risk of decompensation and/or HCC also increased. For instance, a patient with *a* + 10 % increase in LSM values at the 6-month post-treatment, compared to the pretreatment period, experienced *a* + 14 % increase in the hazard of decompensation and/or HCC. This risk escalated further, with *a* + 20 % increase resulting in *a* + 30 % hazard increase, and *a* + 40 % increase leading to *a* + 68 % hazard increase. Finally, when there was *a* + 92 % increase in LSM between the assessed periods, the hazard of decompensation and/or HCC increased to +50 %.

An analysis was conducted to identify the optimal cutoff value in this study concerning the distribution of decompensation and/or HCC curve at the 6-month posttreatment assessment. The presence of an LSM ≥ 19 KPa at the 6-month posttreatment mark was associated with a Hazard Ratio (HR) of 14.533 (95 % CI 6.321 to 36.718) (*p* < 0.001), indicating that patients with an LSM of 19 KPa during this period had a 14.5-fold greater risk of decompensation and/or HCC than did those without such an LSM in the same period.

## Discussion

Presently, with the widespread use of direct-acting antivirals (DAAs), nearly all hepatitis C patients achieve SVR. However, individuals with advanced fibrosis and cirrhosis need continued clinical monitoring for HCC screening and decompensation risk assessment. This underscores the importance of assessing decompensation and/or HCC risk using noninvasive liver fibrosis evaluation methods. In this study, we analyzed the relative variation in the LSM, expressed in KPa through transient elastography, between two clinically significant time points: pre-treatment (visit 1) and 6 months post-treatment (visit 3), when an SVR was established. We observed that a negative relative variation (indicating reduced liver stiffness) was associated with a lower risk of hepatic decompensation and/or HCC, while a positive variation favored adverse outcomes. Moreover, we identified a cutoff value of ≥ 19 KPa at 6 months posttreatment as a predictor of decompensation and/or HCC.

This study followed up patients for 3-years after they achieved SVR and employed three noninvasive methods for assessing liver fibrosis levels, the APRI, FIB4 and particularly transient elastography using FibroScan®. During the pre-treatment period of hepatitis C, noninvasive methods for assessing liver fibrosis are strongly correlated with an accuracy of more than 80 % for patients with mild to moderate fibrosis and more than 98 % for patients with cirrhosis.^19^ However, no established new cutoff values exist for these methods in post-SVR follow-ups, which makes it difficult to compare post-treatment methods. After DAA treatment, the restoration of inflammatory mediators leading to liver improvement is observed. This improvement is reflected in changes in laboratory test values such as transaminases and platelets counts, as well as in noninvasive methods for assessing liver fibrosis, such as the APRI, FIB4 and TE.^20^^,^^21^

When evaluating the APRI and FIB4 scores in this study, these variations may have directly influenced the reduction in the values found at the end of the treatment, since they are methods that include these laboratory parameters in their formulas. This reduction was also demonstrated in the study by Hsu et al.,^22^ who presented an equivalent rate of patients with cirrhosis and a reduction in the APRI score from 1.19 (0.62‒2.43) to 0.48 (0.31‒0.58) and in the FIB4 score from 2.88 (1.56‒5.60) to 2.11 (1.37‒3.76) before pretreatment and end of treatment, respectively. In the present study, the risk of HCC occurrence with these methods was not evaluated. However, the work produced by Ianou et al.^23^ evaluated a FIB4 score ≥ 3.25 and showed a decrease in the annual risk of HCC from 3.8 % in the first year after SVR to 2.4 % in the fourth year of follow-up (*p* = 0.01); in patients with a FIB4 score < 3.25 in the same period, this risk decreased from 1.4 % in the first year to 0.5 % in the fourth year (*p* = 0.01).

Transient elastography with FibroScan® remains the primary method for replacing liver biopsy and has been extensively employed in studies assessing the risk of decompensation and/or HCC in the DAA era. TE showed a statistically significant decrease in the median value expressed in KPa between the pretreatment and end of treatment, but it also demonstrated a continuous decrease in KPa values over this 3-year study, unlike the APRI and FIB4, which did not show the same decrease. As described in the meta-analysis conducted by Singh et al.,^24^ which assessed 24 studies using FibroScan® based TE in the post-SVR follow-up of hepatitis C treatment, this initial reduction was associated with immediate inflammatory improvement following DAAs use, and its long-term continuation could be considered a consequence of liver fibrosis improvement. This ongoing regression of TE was also demonstrated in the study by Knop et al.,^25^ despite having a smaller and more heterogeneous participant group than the present study. Knop et al.'s study showed improvements between the pre-treatment period [median (range), 32.5 (9.1–75) KPa] and the end of treatment [median (range), 21.3 (6.7–73.5) KPa; *p* < 0.001], as well as continued improvement between the end of treatment and the third year [median (range), 16 (4.1–75) KPa; *p* = 0.006].

The variation in TE measurements post-SVR may not occur uniformly among all evaluated patients. Upon close examination, we observed positive, negative, and stable variations in values among the groups based on KPa values (Groups A, B, and C) during the 3-year follow-up period. This find aligns with a similar variability that was also observed in the study conducted by Sporea et al.,^26^ which assessed a cohort of 211 patients. In their study, the mean TE values were 26.4 ± 11.7 KPa with a decrease to 23.5 ± 13.3 KPa (*p* = 0.01); approximately 59.2 % of the patients exhibited a decrease of more than 10 % in KPa values; 24.1 % had stable values; and 16.4 % experienced an increase in KPa values between pretreatment and posttreatment with DAAs. Additionally, in the study by Piecha et al.,^27^ which evaluated 346 patients before and after treatment, 77 % of the patients had a KPa reduction of more than 10 %, 14 % had a reduction of up to 10 %, and 10 % had an increase in KPa greater than 10 %. These variations in responses among patients post-SVR with treatment align with the observation that while there is a decrease in the risk of decompensation and/or HCC following treatment, the risk is not entirely eliminated.

Determining cutoff values through TE for adverse outcomes post-SVR with DAAs is a topic of interest. According to the present analysis, an LSM ≥ 19 KPa at the 6-month posttreatment mark was strongly associated with adverse outcomes, with an HR of 14.5 (95 % CI 6.32 to 36.71; *p* < 0.001). This finding is consistent with those in other studies, such as the one by Vutien et al.,^28^ which analyzed a large cohort of veterans and found that an LSM > 20 KPa in the posttreatment period was independently associated with negative outcomes.

During the era of interferon-based hepatitis treatment, studies demonstrated that patients with advanced fibrosis who achieved SVR had a lower incidence of liver failure and HCC development than untreated patients.^29^, ^30^, ^31^, ^32^

Currently, multiple studies are showing improvements in the occurrence of decompensation and/or HCC with DAA treatment. For example, Kanwal et al.^33^ showed that DAA-treated patients with SVR had a 76 % reduction in the risk of HCC over time. Patients with cirrhosis are the primary population to develop HCC. Mendizabal M et al.^34^ concluded that achieving an SVR reduces the risk of hepatic decompensation (HR = 0.3; 95 % IC 0.1‒0.8; *p* = 0.016) and new-onset HCC (HR = 0.2; 95 % IC 0.1‒0.8; *p* = 0.02). Mathur K et al.^35^ reported an increase in the incidence of HCC among noncirrhotic patients (OR = 1.35, 95 % CI 1.08‒1.69, *p* = 0.009) and a decrease in the incidence of HCC among cirrhotic patients (OR = 0.92, 95 % CI 0.86‒0.98, *p* = 0.012) among those treated with DAAs, resulting in an increase in the rate of HCC resection and a decrease in the rate of liver transplantation. As a result, the guidelines for follow-up of patients who have achieved an SVR and are treated with DAAs recommend that patients classified as F0, F1, and F2 according to noninvasive methods for assessing liver fibrosis may discontinue follow-up after achieving an SVR. There is some divergence in the international guidelines for patients classified as F3 and F4. The European guidelines recommend that F3 and F4 patients undergo surveillance abdominal ultrasound every 6-months after SVR, while the American guideline recommends follow-up only for patients with cirrhosis or F4.^8^^,^^36^

In this study, the predictive value of each variable at baseline (gender, age, lifestyle, diabetes, hypertension, and others) for the dependent time-based outcome of decompensation and/or HCC was tested. The relative change in FibroScan® measurements was the best predictor of negative outcomes and/or HCC in this sample. The relative change in liver stiffness occurring between the pretreatment and 6-months posttreatment visits had an HR of 1.03; 95 % IC 1.003; 1.021; *p* = 0.014. This finding allowed for the stratification of HR, where patients with a negative change of −40 % had a −41 % reduction in the hazard of decompensation and/or HCC. When this reduction was only −10 %, the hazard was reduced by −12 %. Conversely, when this relative change was positive (indicating an increase in liver rigidity assessed by FibroScan®), the risk of negative outcomes increased accordingly. For instance, a patient with *a* + 50 % increase in relative change had *a* + 92 % increase in the hazard of decompensation and/or HCC. When there was an increase of +10 % in the LSM variation between the pretreatment and the 6th month posttreatment, there was *a* + 12 % increase in the hazard of negative outcomes. Ravaioli et al.^9^ also analyzed the correlation between the variation in elastography values and the occurrence of HCC. They followed up with 135 patients for a period of 15-months and found a significant reduction in elastography values in both patients who developed HCC and those who did not. However, the reduction was significantly smaller in the patients who developed HCC (−18.0 % vs. −28.9 %, *p* = 0.005). In contrast, in the present study, a broader stratification of hazards that patients may experience with the variations presented by elastography was conducted. In the study by Semmler et al.,^37^ which also addressed the variation in LSM in patients with LSM ≥ 10.0 KPa, it was reported that a 20 % increase in LSM dynamics is associated with a 50 % increase in the risk of decompensation, while a 20 % decrease leads to a 50 % reduction in the risk of decompensation. When focusing solely on the 369 patients who achieved SVR in the context of hepatitis C (HR = 2.16, 95 % CI 1.76‒2.62; *p* < 0.01), a 20 % increase in the average LSM variation over 180-days was associated with this outcome. The difference in the initial LSM between the present study (LSM ≥ 9.5 KPa) and that of Semmler et al.^37^ (LSM ≥ 10.0 KPa) may have contributed to the lower risk ratio of decompensation observed: 33 % versus 50 % for a positive variation of +20 % or a decrease of −20 %, respectively, with 25 % versus 50 %.

This study has several limitations, such as the relatively small number of included patients, which resulted in a limited number of negative outcomes for analysis. The COVID-19 pandemic delayed the third-year FibroScan® evaluation for some patients, thereby extending the evaluation period. On the other hand, this was a prospective study involving systematic TE evaluation over a period of more than 3-years, conducted by a single experienced operator, which eliminated the variability in acquisitions that can occur between different operators.

In conclusion, this study demonstrated that in patients with hepatitis C, performing liver elastography assessments before treatment and at 6-months posttreatment can provide relevant prognostic information. For patients initially present with advanced fibrosis and cirrhosis (LSM ≥ 9.5 KPa), continuous monitoring for hepatic decompensation and HCC is generally recommended. Additionally, we found that the difference in the relative changes between these two TE measurements was related to the occurrence of these negative outcomes.

## Authors’ contributions

Pessoa MG conceived and designed the study, contributed to the data analysis and interpretation, and wrote and reviewed the manuscript. Ragazzo TG collected and assembled the data, provided care for the patients, contributed to the analysis and interpretation, and wrote the manuscript. Carrilho FJ, Oliveira CP, Mazo DF, Zitelli PMY provided care for the patients and critically reviewed the manuscript. All the authors critically revised the manuscript, approved the final version to be published, and agreed to be accountable for all aspects of the work.

## Funding

This research did not receive any specific grant from funding agencies in the public, commercial, or not-for-profit sectors.

## Conflicts of interest

The authors declare no conflicts of interest.
